# Assessing Risk of Early ICU Admission or Death at Emergency Department Triage: Clinical Judgment Versus Early Warning Scores

**DOI:** 10.1111/acem.70377

**Published:** 2026-07-16

**Authors:** Nicola Bonadia, Piergiacomo Maria Cacciamani Fanelli, Davide Antonio Della Polla, Valeria Maccauro, Giuseppe De Matteis, Andrea Piccioni, Antonio Gasbarrini, Claudio Sandroni, Francesco Franceschi, Marcello Covino

**Affiliations:** ^1^ Emergency Department Fondazione Policlinico Universitario A. Gemelli IRCCS Rome Italy; ^2^ Department of Geriatrics Fondazione Policlinico Universitario A. Gemelli IRCCS Rome Italy; ^3^ Department of Internal Medicine and Gastroenterology Fondazione Policlinico Universitario A. Gemelli IRCCS Rome Italy; ^4^ Università Cattolica del Sacro Cuore Rome Italy; ^5^ Department of Anesthesiology and Intensive Care Medicine Fondazione Policlinico Universitario A. Gemelli IRCCS Rome Italy

## Abstract

**Background:**

Early Warning Scores (EWS) are widely used to standardize the identification of clinical deterioration, yet their comparative performance against clinical judgment in Emergency Department (ED) triage remains uncertain. We aimed to evaluate whether commonly used EWS match or outperform clinical judgment in predicting early adverse outcomes.

**Methods:**

We conducted a retrospective observational study including 361,927 adult ED presentations at a tertiary‐care academic center from 2015 to 2024. Clinical judgment was operationalized as the triage category assigned at the initial evaluation. Five EWS (NEWS, NEWS2, MEWS, REMS, and ViEWS) were computed using vital signs recorded at ED presentation. The primary outcome was a composite of 24‐h mortality or intensive care unit (ICU) admission. Discrimination, calibration, decision curve analysis, and reclassification metrics were used to compare models.

**Results:**

The primary outcome occurred in 1.17% of patients. Discriminatory performance was highest for ViEWS (AUC 0.875) and clinical judgment (AUC 0.872), with no significant difference between them. Other EWS demonstrated significantly lower AUCs. In precision–recall and threshold‐based analyses, clinical judgment maintained higher specificity at higher‐risk thresholds while preserving adequate sensitivity. Decision curve analysis showed comparable or greater net benefit for clinical judgment than for all tested EWS across clinically relevant thresholds. Reclassification metrics showed no improvement with EWS over clinical judgment.

**Conclusion:**

Commonly used EWS did not clearly outperform triage clinical judgment in predicting early ICU admission or death. These findings support further investigation of how structured scores and clinical judgment may provide complementary information, particularly in identifying patients whose risk may be modified by timely escalation of care.

## 1 Introduction

Early recognition of clinical deterioration is a key objective of emergency and acute care systems. Physiological abnormalities often precede cardiac arrest, intensive care unit (ICU) admission, and death, providing an opportunity for early intervention [1, 2, 3].

Early Warning Scores (EWS) were developed to standardize detection of physiological deterioration and trigger escalation of care [4]. Systems such as the National Early Warning Score (NEWS) and its updated version NEWS2 combine vital sign abnormalities into a numerical risk estimate linked to escalation protocols [5, 6]. Several studies have shown that these scores can predict short‐term deterioration in Emergency Department (ED) populations, although their performance varies across settings, outcomes, and decision thresholds [7, 8, 9, 10, 11, 12, 13].

However, the clinical value of EWS ultimately depends on their performance relative to existing clinical practice. In routine ED care, experienced providers rapidly integrate vital signs with contextual clinical information, including presenting symptoms, observed distress, and overall appearance, to identify patients requiring urgent attention. In the Emergency Department, this assessment is partly formalized through triage procedures, which represent a semi‐structured form of clinical judgment integrating physiological measurements with contextual clinical information. Evidence comparing structured scoring systems with clinical judgment in the ED setting remains limited and inconsistent. Some studies suggest comparable predictive performance, whereas others suggest that clinical assessment may better identify patients requiring intensive care or urgent escalation [14, 15]. Accordingly, although the predictive performance of EWS has been widely studied and different scores have been compared across settings [16, 17, 18, 19, 20, 21], it remains unclear whether EWS outperform clinical judgment during ED triage.

To address this gap, we retrospectively compared the predictive performance of commonly used Early Warning Scores with triage assessment by experienced triage nurses for identifying ED patients at risk of early clinical deterioration, defined as death or ICU admission within 24 h of presentation.

## 2 Materials and Methods

### 2.1 Study Design and Setting

We conducted a retrospective observational study at the Emergency Department (ED) of the Fondazione Policlinico Universitario A. Gemelli IRCCS, a tertiary‐care referral center in Rome, Italy. The study period extended from January 1, 2015, to December 31, 2024.

### 2.2 Selection of Participants

All consecutive adult non‐pregnant patients (≥ 18 years) presenting to the Emergency Department during the study period were considered eligible for inclusion. Patients were included if no more than two physiological variables required for the calculation of Early Warning Scores were missing at the time of the initial ED assessment. We excluded from the final analysis patients for whom formal risk stratification was unlikely to provide meaningful support to triage decisions. Moreover, we excluded from the analysis patients whose condition mandated early ICU admission according to institutional protocols (e.g., ST‐elevation myocardial infarction or acute stroke eligible for reperfusion therapy).

Specifically, patients who presented at least one of the following criteria were excluded:

1. Patients with an apparent extreme critical condition:
  - Patients who arrived at ED already intubated by the Emergency Medical Services.
  - Those already in cardiac arrest or with evident impending cardiac arrest.
2. Patients who died or were admitted to Intensive Care Unit (ICU) within the first hour after ED arrival.
3. Trauma patients.
4. Female patients who were pregnant at the time of ED arrival or whose pregnancy was discovered during ED stay.

**FIGURE 1:**
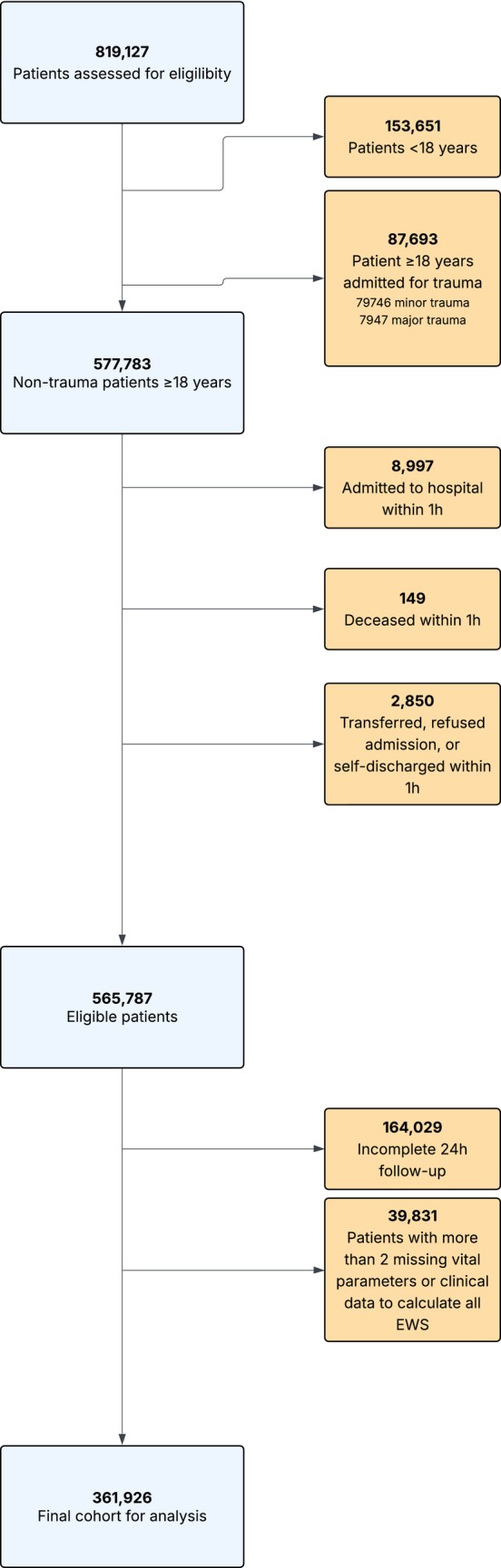
Enrolling flow chart of the patients included in the study. In 18,843 cases (5.21% in the study cohort) with less than two missing parameters for EWS calculation, imputation was performed.

A flow chart of enrollment is shown in Figure 1.

### 2.3 Measurements

Demographic characteristics (age and sex), vital signs, and clinical variables were extracted from electronic medical records. Physiological parameters collected at ED presentation included heart rate, respiratory rate, systolic and diastolic blood pressure, mean arterial pressure, peripheral oxygen saturation, body temperature, and Glasgow Coma Scale score. Additional clinical variables included oxygen supplementation and selected presenting symptoms.

Relevant data were collected from the ED Electronic Health Record system, which is mandatory for all EDs at the regional level. This system allows for registration of demographic characteristics and vital signs both during triage and at subsequent re‐evaluations, for the attribution of a final triage code, and for the registration of the final disposition or outcome. Data were automatically collected from the backend database by one of the authors (MC). No manual validation was performed, but data were evaluated for consistency before the final analysis (i.e., outliers or unrealistic values).

#### 2.3.1 Clinical Judgment

Clinical judgment at presentation was operationalized using the triage severity category assigned by the triage nurse during the initial patient evaluation. Patients were classified into three urgency levels: Emergency, Urgency, and Non‐Urgency. Triage assignment represents a semi‐structured clinical assessment in which experienced providers integrate physiological measurements with contextual clinical information, including presenting symptoms, patient appearance, and overall clinical impression. Nurses are required to complete a formal certification process before performing triage. The triage system in use at our institution is developed at the regional level [22]. It requires recognition of specific time‐critical clinical syndromes (e.g., ST‐elevation myocardial infarction or stroke) and emphasizes both the interpretation of vital signs in the context of the overall clinical picture and the value of nurses' expert judgment. This triage system allows for five distinct urgency codes, which range from the most (code 1) to the least (code 5) urgent. However, this system applies codes 3, 4, and 5 to patients whose condition does not present risk of deterioration, with the main difference between them consisting of the anticipated complexity of management. Consequently, for the purpose of this analysis, codes 3, 4, and 5 were collapsed into the Non‐Urgent category, while code 2 has been redefined as Urgency and code 1 as Emergency.

#### 2.3.2 Early Warning Scores

The following prognostic tools were calculated for each patient, independently of Clinical Judgment: National Early Warning Score (NEWS) [5], National Early Warning Score 2 (NEWS2) [6], Modified Early Warning Score (MEWS) [23], Rapid Emergency Medicine Score (REMS) [24], and VitalPAC Early Warning Score (ViEWS) [25]. All scores were calculated using physiological variables recorded at Emergency Department presentation, in accordance with their original published definitions. Scores requiring AVPU or ACVPU (MEWS and NEWS/NEWS2) used the directly recorded AVPU/ACVPU value, while scores requiring GCS (REMS and ViEWS) used the directly recorded GCS value. For NEWS2, patients with a confirmed history of COPD were scored using SpO2 Scale 2; all remaining patients were scored using Scale 1, consistent with standard practice at initial ED assessment.

### 2.4 Outcomes

The primary outcome was a composite endpoint of all‐cause mortality or admission to the intensive care unit (ICU) within 24 h of ED presentation. Secondary outcomes were the individual components of the primary composite outcome, evaluated separately.

### 2.5 Analysis

Continuous variables are reported as means with standard deviations or medians with interquartile ranges, as appropriate. Categorical variables are presented as counts and percentages.

Among the 3.7% of patients in the eligible cohort with one or two missing physiological parameters, multiple imputation was performed under a missing‐at‐random (MAR) assumption using the Fully Conditional Specification (FCS) method implemented in SPSS version 25 (IBM Corp., Armonk, NY, USA). FCS is an iterative Markov chain Monte Carlo chained‐equations procedure in which each incomplete variable is imputed from a regression model conditioned on all other variables. Each affected variable (heart rate, respiratory rate, systolic and diastolic blood pressure, mean arterial pressure, peripheral oxygen saturation, body temperature, and Glasgow Coma Scale score) had less than 5% missing values. All available variables, excluding outcome variables, were used for imputation. Five imputed datasets were generated, and for each missing value, the average of the corresponding imputed values across the five datasets was used to create a single completed dataset for the final analysis. Imputed values were constrained within the observed range for each parameter. No formal sensitivity analysis was performed because of the very low proportion of missing data.

Score and Clinical Judgment performance were assessed in terms of discrimination and calibration. Discrimination refers to the ability to distinguish between patients with and without the outcome, while calibration reflects the agreement between predicted and observed event probabilities.

Discriminatory performance of early warning scores and Clinical Judgment was evaluated using receiver‐operating‐characteristic (ROC) curves, with calculation of the area under the curve (AUC) and corresponding 95% confidence intervals. Comparisons between AUCs were performed using the DeLong method. Calibration was assessed using Brier scores, with 95% confidence intervals and *p*‐values derived from bootstrap resampling, and results were compared to both a null model and the best‐performing score. Since all Early Warning Scores and Clinical Judgment assessments were applied to the same patients, all comparisons were conducted within a paired framework. DeLong's test accounts for within‐subject correlation by design, and Brier scores were bootstrapped at the patient level to preserve this paired structure across scores. Additionally, calibration plots illustrating observed versus predicted probabilities were generated for each EWS, with calibration slopes derived from logistic regression models including each EWS as the sole predictor.

Model calibration was assessed graphically using calibration plots and analytically by comparing observed and predicted risks across risk strata by the CITL (Calibration‐in‐the‐Large) metric. Clinical utility was evaluated using decision curve analysis, estimating the net benefit of each score across a range of clinically relevant threshold probabilities. Incremental prognostic value of early warning scores compared with Clinical Judgment was further assessed using reclassification metrics, including the net reclassification improvement (NRI) and integrated discrimination improvement (IDI).

All analyses were performed using SPSS 25 (IBM Corp., Armonk, NY, USA) and custom Python programming (Python version 3.12.2), using the following libraries for statistical analysis and figure generation: pandas, NumPy, SciPy, scikit‐learn, statsmodels, Matplotlib, and seaborn.

A two‐sided *p*‐value of less than 0.05 was considered statistically significant in all the analyses.

### 2.6 Ethical Statement

This study has been approved by the local Ethics Committee (IRB authorization #0025817/22) and has been performed according to the ethical standards established in the 1964 Declaration of Helsinki and its later amendments [26]. Since the patients' data were anonymized, the informed consent was waived. Clinical trial ID NCT05593692.

## 3 Results

### 3.1 Characteristics of Study Subjects

Overall, 361,927 adult Emergency Department presentations were analyzed (Figure 1). The primary composite outcome of death or intensive care unit admission within 24 h occurred in 4238 cases (1.17%).

Demographic and clinical characteristics of the cohort are reported in Table 1, stratified by outcome.

**TABLE 1:** Summary statistics of physiological and demographic variables.

Variable	All cases	No outcome	Deceased 24 h	ICU 24 h	Deceased and ICU 24 h	Deceased or ICU 24 h
*N*	361,927 (361927)	98.83% (357689)	0.40% (1452)	0.80% (2880)	0.03% (94)	1.17% (4238)
Age	55 (39–73)	55 (39–72)	81 (71–87)	68 (55–78)	74 (62–79)	73 (60–82)
Sex (Male)	48.07% (173993)	47.99% (171652)	48.14% (699)	58.92% (1697)	58.51% (55)	55.24% (2341)
Clinical judgment						
Emergency	4.16% (15058)	3.51% (12537)	52.82% (767)	62.99% (1814)	63.83% (60)	59.49% (2521)
Urgency	27.84% (100763)	27.81% (99463)	35.95% (522)	27.74% (799)	22.34% (21)	30.67% (1300)
Non‐Urgency	68.00% (246106)	68.69% (245689)	11.23% (163)	9.27% (267)	13.83% (13)	9.84% (417)
Early warning score						
NEWS	0 (0–3)	0 (0–2)	6 (4–9)	5 (3–7)	7 (5–10)	5 (3–8)
MEWS	0 (0–2)	0 (0–2)	3 (2–5)	2 (2–4)	3 (2–5)	3 (2–4)
REMS	3 (0–6)	3 (0–6)	9 (7–11)	7 (5–9)	8 (6–10)	8 (6–10)
VIEWS	0 (0–3)	0 (0–3)	7 (4–10)	6 (4–8)	7 (5–10)	6 (4–9)
NEWS2	0 (0–3)	0 (0–3)	6 (4–9)	5 (3–7)	7 (5–9)	5 (3–7)
Chief complaint						
Dyspnea	9.54% (34514)	9.25% (33078)	38.98% (566)	31.46% (906)	38.30% (36)	33.88% (1436)
Chest pain	7.89% (28569)	7.94% (28416)	4.13% (60)	3.37% (97)	4.26% (4)	3.61% (153)
Abdominal pain	14.65% (53024)	14.75% (52747)	9.99% (145)	4.93% (142)	10.64% (10)	6.54% (277)
Diarrhea	3.13% (11315)	3.12% (11174)	4.13% (60)	2.95% (85)	4.26% (4)	3.33% (141)
Emesis	7.38% (26709)	7.38% (26382)	9.16% (133)	7.08% (204)	10.64% (10)	7.72% (327)
Syncope	4.78% (17308)	4.79% (17124)	5.10% (74)	3.96% (114)	4.26% (4)	4.34% (184)
Vertigo	3.30% (11959)	3.34% (11946)	0.14% (2)	0.38% (11)	0.00% (0)	0.31% (13)
Suppl_O2	2.80% (10117)	2.55% (9136)	28.37% (412)	20.49% (590)	22.34% (21)	23.15% (981)
Type2_RF_Risk	1.96% (7091)	1.90% (6785)	6.89% (100)	7.40% (213)	7.45% (7)	7.22% (306)
Oliguria	0.89% (3235)	0.87% (3125)	4.34% (63)	1.63% (47)	0.00% (0)	2.60% (110)
Vital parameters						
HR (beats/min)	83 (74–94)	83 (74–93)	90 (74–112)	90 (77–110)	96 (80–121)	90 (76–110)
RR (rate/min)	15 (12–20)	15 (12–20)	20 (15–23)	19 (14–23)	23 (20–24)	19 (14–23)
SBP (mmHg)	134 (120–150)	135 (120–150)	113 (90–135)	130 (110–150)	120 (100–140)	125 (100–145)
DBP (mmHg)	80 (70–90)	80 (70–90)	67 (54–80)	76 (60–89)	70 (60–84)	73 (60–85)
MAP (mmHg)	98.0 (88.3–108.3)	98.0 (88.3–108.3)	83.3 (66.7–98.0)	93.7 (79.3–107.7)	87.8 (73.3–102.8)	90.7 (73.7–105.0)
SaO2 (%)	97 (95–99)	97 (95–99)	92 (86–96)	95 (89–98)	92 (85–96)	94 (88–98)
Body Temp (°C)	36.0 (36.0–38.0)	36.0 (36.0–38.0)	36.1 (36.0–38.0)	36.0 (36.0–38.0)	36.0 (36.0–36.8)	36.1 (36.0–38.0)
GCS	13 (8–15)	13 (8–15)	14 (8–15)	14 (9–15)	15 (15–15)	14 (8–15)

*Note:* Metric variables and EWS scores are reported as median (Q1–Q3); nominal and ordinal values are reported as percentage (number of cases).

Patients who experienced the primary outcome were older and predominantly male. Frequency distributions of clinical features recorded at presentation are shown in Table 1. Higher EWS values and higher Clinical Judgment categories were associated with the primary outcome.

### 3.2 Distribution of Risk Scores by Outcome

**FIGURE 2:**
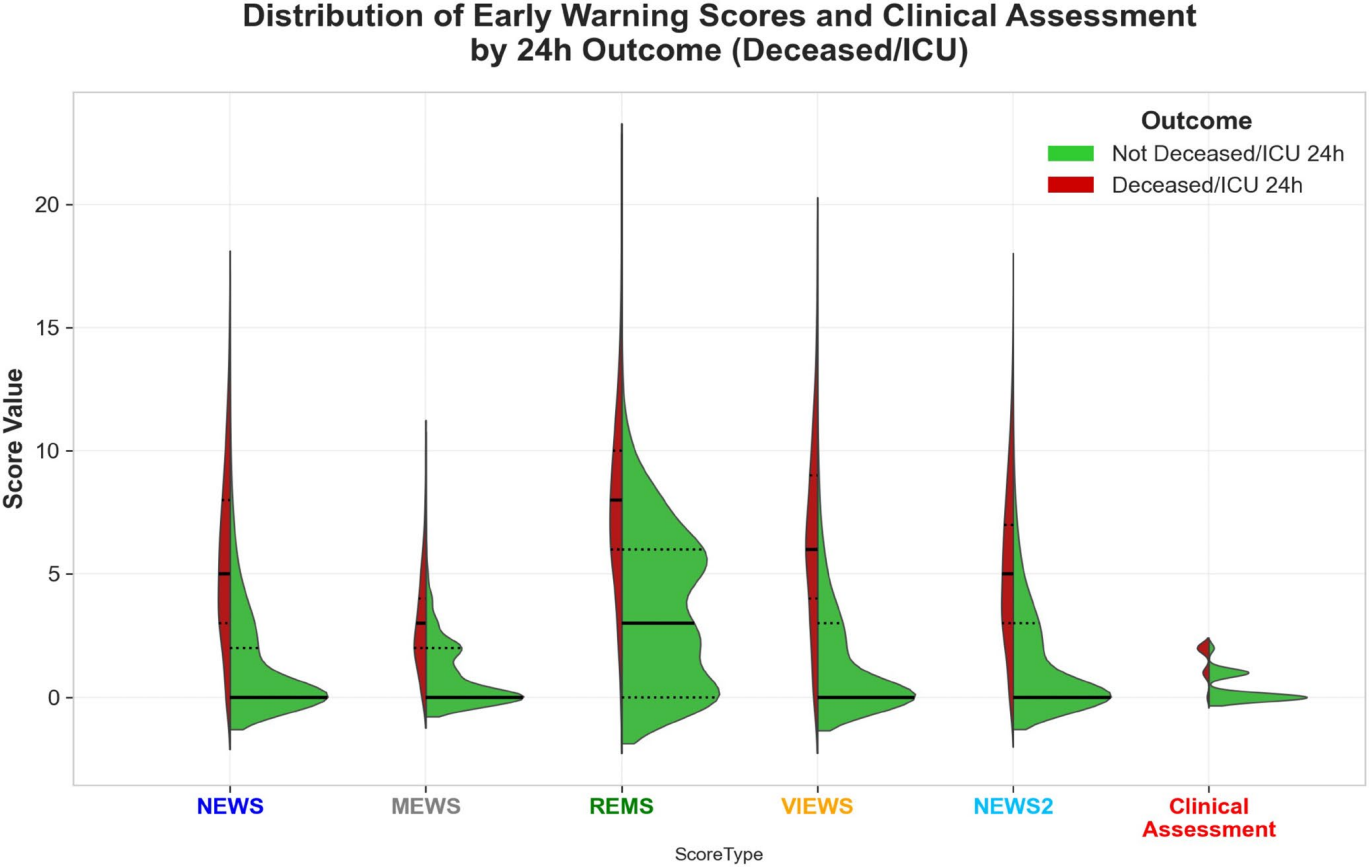
Violin plots showing the distribution of risk score values (NEWS, MEWS, REMS, VIEWS, NEWS2, Clinical Assessment) stratified by 24‐h outcome (Deceased/ICU vs. Not Deceased/ICU). The solid black lines indicate the median, and the dotted lines represent the first (Q1) and third (Q3) quartiles of the score distributions.

Distributions of Early Warning Scores and Clinical Judgment stratified by 24‐h outcome status are presented in Figure 2. Overall, score values were shifted toward higher ranges among patients who died or required ICU admission compared with those who did not experience the outcome, with both median and upper quartile values consistently higher in the outcome group across all scoring systems. Some overlap between distributions was observed at lower score values for several models.

### 3.3 Discriminatory Performance

**FIGURE 3:**
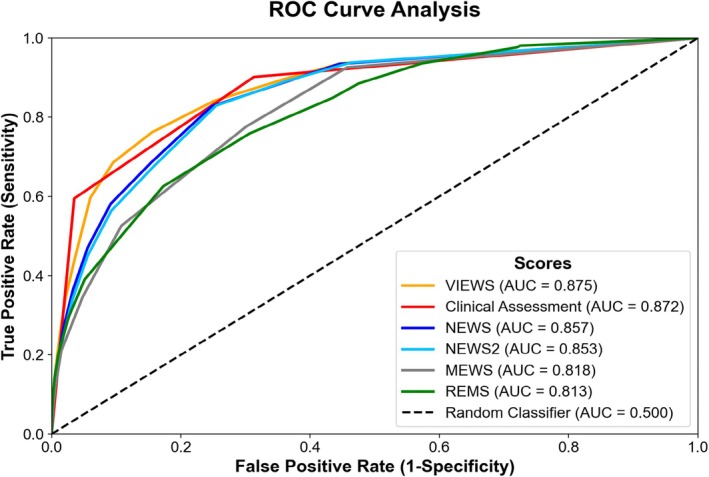
Receiver Operating Characteristic (ROC) curve analysis comparing the predictive performance of NEWS, NEWS2, REMS, MEWS, VIEWS, and clinical assessment for 24‐h outcomes (Deceased/ICU).

Receiver operating characteristic analysis results are shown in Figure 3. The highest area under the curve was observed for ViEWS (AUC 0.875, 95% CI 0.869–0.881), followed by Clinical Judgment (AUC 0.872, 95% CI 0.866–0.877). The difference in AUC between ViEWS and Clinical Judgment was not statistically significant. Compared with Clinical Judgment, NEWS, NEWS2, MEWS, and REMS showed lower AUC values, with differences in AUC reaching statistical significance.

**FIGURE 4:**
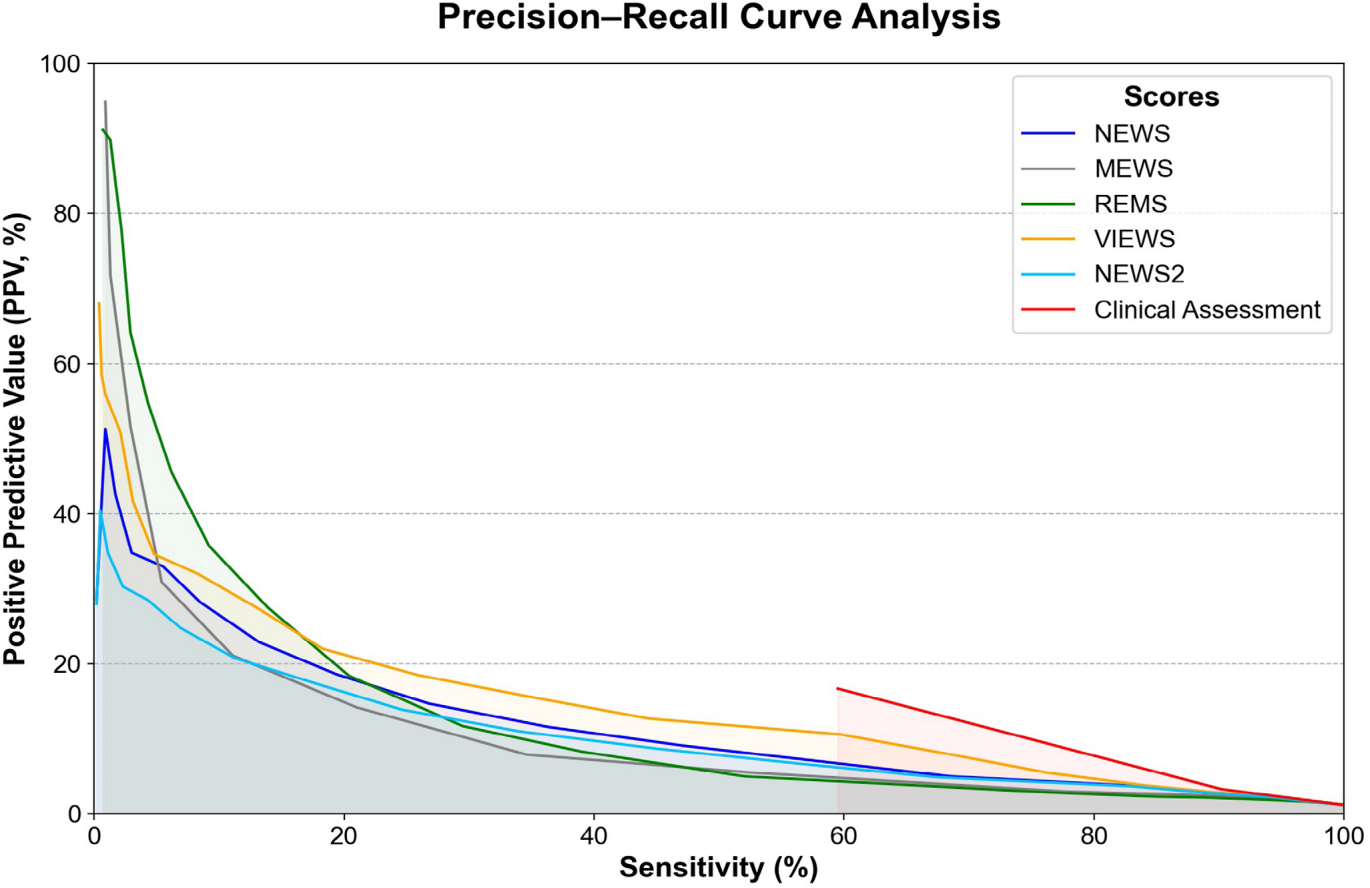
Precision–recall curves comparing early warning scores (NEWS, MEWS, REMS, VIEWS, NEWS2) and clinical assessment. Positive predictive value (PPV) declines with increasing sensitivity across all methods, illustrating trade‐offs in discriminative performance and highlighting differences in predictive efficiency among scoring systems.

The precision–recall curves are shown in Figure 4. As expected in a cohort with low prevalence of the primary outcome, positive predictive value decreased as sensitivity increased for all models. Variability among models was observed within clinically relevant sensitivity ranges, and both Clinical Judgment and ViEWS demonstrated higher precision at comparable sensitivity levels compared with other Early Warning Scores. The Clinical Assessment curve is truncated at ~60% sensitivity due to its 3‐level ordinal scale (0, 1, 2). Because the strictest threshold (score of 2) already identifies 60% of events, the lack of higher ordinal values precludes evaluation at lower sensitivity intervals, distinguishing it from higher‐granularity scores that approach zero sensitivity.

Threshold‐based performance metrics at prespecified probability cut‐offs are summarized in Table 2. At lower positive predictive value (PPV) targets, sensitivity estimates were higher and specificity estimates were lower across all models. At intermediate and higher PPV thresholds, Clinical Judgment showed higher PPV than several Early Warning Scores at comparable threshold levels.

**TABLE 2:** Diagnostic performance of early warning scores and clinical assessment at thresholds calibrated to achieve predefined positive predictive value (PPV) targets (≥ 2.5%, ≥ 5%, ≥ 10%, and ≥ 25%) for the study outcome.

PPV target (%)	Score	Threshold	PPV [95% CI]	NPV [95% CI]	Sensitivity [95% CI]	Specificity [95% CI]
≥ 2.5%	NEWS	2	2.9 [2.8–3.0]	99.8 [99.8–99.8]	88.6 [88.0–89.2]	64.4 [63.8–64.9]
≥ 2.5%	MEWS	2	3.0 [2.9–3.1]	99.6 [99.6–99.6]	77.7 [77.1–78.3]	69.9 [69.3–70.5]
≥ 2.5%	REMS	6	2.9 [2.8–3.0]	99.6 [99.6–99.6]	75.9 [75.2–76.5]	69.4 [68.8–70.0]
≥ 2.5%	VIEWS	2	2.9 [2.8–3.0]	99.8 [99.8–99.8]	89.6 [89.0–90.1]	64.1 [63.5–64.6]
≥ 2.5%	NEWS2	2	2.8 [2.7–2.9]	99.8 [99.8–99.8]	89.2 [88.6–89.8]	63.5 [63.0–64.1]
≥ 2.5%	Clinical Judgment	1	3.3 [3.2–3.4]	99.8 [99.8–99.8]	90.2 [89.6–90.7]	68.7 [68.1–69.3]
≥ 5%	NEWS	4	5.0 [4.8–5.2]	99.6 [99.5–99.6]	68.5 [68.0–69.1]	84.6 [84.0–85.2]
≥ 5%	MEWS	3	5.5 [5.2–5.7]	99.4 [99.3–99.4]	52.6 [52.0–53.2]	89.2 [88.6–89.8]
≥ 5%	REMS	8	5.0 [4.8–5.2]	99.4 [99.3–99.4]	52.0 [51.4–52.6]	88.2 [87.6–88.8]
≥ 5%	VIEWS	4	5.5 [5.3–5.7]	99.7 [99.6–99.7]	76.2 [75.6–76.8]	84.5 [83.9–85.0]
≥ 5%	NEWS2	5	6.7 [6.5–7.0]	99.4 [99.4–99.5]	56.4 [55.8–56.9]	90.8 [90.2–91.3]
≥ 5%	Clinical Judgment	2	16.7 [16.2–17.3]	99.5 [99.5–99.5]	59.5 [58.9–60.1]	96.5 [95.9–97.1]
≥ 10%	NEWS	7	11.6 [11.1–12.1]	99.2 [99.2–99.3]	36.3 [35.7–36.8]	96.7 [96.2–97.3]
≥ 10%	MEWS	5	14.2 [13.4–15.1]	99.1 [99.0–99.1]	21.0 [20.4–21.6]	98.5 [97.9–99.1]
≥ 10%	REMS	10	11.7 [11.1–12.3]	99.1 [99.1–99.2]	29.5 [28.9–30.2]	97.4 [96.7–98.0]
≥ 10%	VIEWS	6	10.6 [10.2–10.9]	99.5 [99.5–99.5]	59.7 [59.1–60.2]	94.0 [93.4–94.6]
≥ 10%	NEWS2	7	11.0 [10.5–11.6]	99.2 [99.2–99.2]	34.0 [33.5–34.6]	96.8 [96.2–97.3]
≥ 10%	Clinical Judgment	2	16.7 [16.2–17.3]	99.5 [99.5–99.5]	59.5 [58.9–60.1]	96.5 [95.9–97.1]
≥ 25%	NEWS	11	28.2 [25.8–30.7]	98.9 [98.9–99.0]	8.5 [8.0–9.1]	99.7 [99.2–100]
≥ 25%	MEWS	7	30.9 [27.6–34.3]	98.9 [98.9–98.9]	5.4 [4.8–6.0]	99.9 [99.3–100]
≥ 25%	REMS	12	27.5 [25.6–29.4]	99.0 [99.0–99.0]	13.9 [13.3–14.5]	99.6 [98.9–100]
≥ 25%	VIEWS	11	27.4 [25.5–29.4]	99.0 [98.9–99.0]	13.1 [12.5–13.7]	99.6 [99.0–100]
≥ 25%	NEWS2	16	28.0 [14.3–47.6]	98.8 [98.8–98.9]	0.2 [0–0.7]	100.0 [99.4–100]

*Note:* For each scoring system (NEWS, MEWS, REMS, VIEWS, NEWS2) and for clinical assessment, the score threshold required to reach the target PPV is reported together with the resulting PPV, negative predictive value (NPV), sensitivity, and specificity, with corresponding 95% confidence intervals.

### 3.4 Calibration

Calibration plots demonstrated close alignment between predicted and observed probabilities across the range of estimated risks, as shown in Figure S1. Predicted risks were compared with observed event rates across increasing risk strata for each model. Calibration‐in‐the‐large estimates were close to zero for all models, and calibration slope values were close to unity, with no statistically significant differences between Early Warning Scores and Clinical Judgment, as reported in Table S1. The Brier score analysis indicated that all models performed significantly better than the null model, suggesting that they provide meaningful predictive information beyond prevalence alone. Clinical Judgment had the lowest Brier score, which represents the best overall probabilistic accuracy, as shown in Table S2.

### 3.5 Clinical Utility and Decision Curve Analysis

**FIGURE 5:**
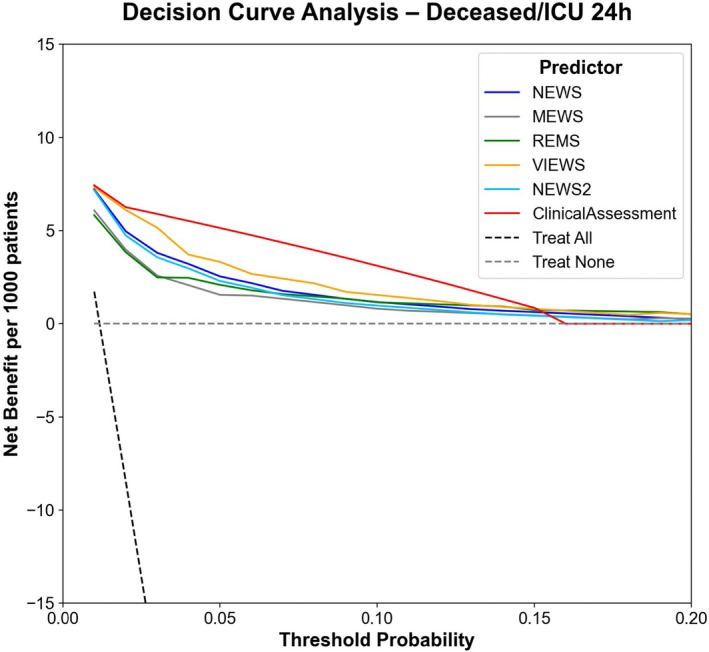
Decision curve analysis for 24‐h mortality or ICU admission. Net benefit per 1000 patients is shown across threshold probabilities for early warning scores and clinical assessment, compared with treat‐all and treat‐none strategies.

Net benefit estimates were evaluated across a range of threshold probabilities relevant to Emergency Department decision‐making. Clinical Judgment and ViEWS showed similar net benefit profiles across a wide span of thresholds, while MEWS and REMS demonstrated consistently lower net benefit values compared with Clinical Judgment and ViEWS for most threshold probabilities. Decision curve metrics for all models are presented in Figure 5.

### 3.6 Reclassification Analysis

Net reclassification improvement estimates were negative for Early Warning Scores when compared with Clinical Judgment for both outcome events and non‐events. None of the reclassification metrics indicated a statistically significant improvement over Clinical Judgment, as shown in Table S3. The relative performances of Clinical Judgment and Early Warning Scores at a realistic specificity threshold are presented in Figures S2 and S3.

### 3.7 Secondary Endpoints

When considered separately, ICU admission within 24 h and death within 24 h showed different performance patterns. Clinical Judgment outperformed all Early Warning Scores (EWS) in predicting ICU admission within 24 h, whereas it was inferior to all EWS, with the sole exception of MEWS, in predicting 24‐h mortality (Tables S4 and S5).

## 4 Discussion

In this large cohort of Emergency Department patients, Early Warning Scores showed discrimination comparable to trained triage nurse judgment but did not show superior predictive ability for early ICU admission or mortality.

Although initially developed as physiological track‐and‐trigger systems to detect evolving physiological deterioration [4], their role has progressively expanded beyond longitudinal monitoring to include their application as prediction tools [27]. In contemporary Emergency Department practice, these tools are frequently used as pragmatic single‐time‐point risk stratification tools during the initial triage assessment.

The appeal of structured risk prediction tools in this setting is intuitive. ED teams operate under time pressure, high patient volume, and substantial uncertainty, and reproducible decision aids may support consistent resource allocation and patient prioritization.

However, the clinical value of any prediction rule rests, ultimately, on its ability to improve existing decision processes [28, 29]. In ED triage, these processes are inherently semi‐structured, combining physiological measurements with contextual cues such as presenting symptoms, observed distress, baseline functional status, and anticipated clinical trajectories. While structured rules may compensate for limited experience or reduce variability, some authors have argued that subjectivity is not eliminated but rather shifted to the decision of whether to apply the rule in a given patient [30].

Evidence comparing structured scoring systems with clinical judgment in acute care remains heterogeneous. Although standardized tools may enhance sensitivity in some settings, several studies have shown comparable or even superior performance of clinical gestalt [31, 32, 33, 34], particularly when decisions involve complex contextual information. Systematic reviews have therefore highlighted the limited evidence supporting replacement of clinical judgment with Early Warning Scores in prehospital and Emergency Department environments [15, 35]. For example, MEWS has been reported to offer higher sensitivity than clinical gestalt in pre‐hospital settings, although with reduced specificity [14]. Conversely, a recent Danish study found that a simple, unstructured “eyeball” judgment outperformed a structured decision system within ED triage operations [36].

In our study, we compared trained triage nurse judgment with commonly used EWS across multiple performance dimensions, including discrimination, calibration, clinical utility, and reclassification. Discrimination was broadly similar, with ViEWS showing the highest AUROC, although not significantly higher than clinical judgment (Figure 3). However, relevant differences emerged when analyses focused on decision‐relevant regions of the risk spectrum. In this low‐prevalence setting, precision–recall curves and threshold‐based evaluations revealed a more favorable balance between sensitivity and false positives for clinical judgment (Figure 4). At lower positive predictive value thresholds, clinical judgment maintained high sensitivity with specificity comparable to the best performing EWS, while at higher thresholds it achieved markedly greater specificity. These findings suggest that the coarser categorization inherent to clinical judgment may improve specificity near critical decision points, whereas the finer gradation of EWS may provide additional discrimination among intermediate‐risk patients. This pattern supports the interpretation that structured scores can complement rather than replace clinical judgment.

Decision curve analysis further reinforced this interpretation (Figure 5). Clinical judgment provided equal or greater net benefit across most clinically relevant threshold probabilities, and reclassification analyses demonstrated no improvement when EWS were added to clinical judgment alone. Most misclassifications involved non‐events, reflecting the low prevalence of adverse outcomes in our cohort. These findings may partially reflect the intended goals of the NEWS family of scores, which were developed for ward populations to guide monitoring intensity and reassessment frequency, and were therefore optimized for finer stratification within intermediate‐risk ranges rather than for predicting the relatively infrequent critical outcomes in the Emergency Department.

Calibration metrics, assessed through the Brier score, revealed no clinically meaningful difference between EWS and clinical judgment. Given the large cohort and the low prevalence of events, small numerical variations at the third decimal place are unlikely to represent meaningful performance distinctions.

Several mechanisms might explain the observed patterns. One possible explanation is that triage decisions can both shape and anticipate subsequent care. This process can operate directly, by influencing ICU admissions, or indirectly, through trust‐based interactions, shared informal practices, and by internalizing downstream decision patterns. Thus, triage decisions could partly influence the very outcomes that they are intended to predict [37]. Our finding that clinical judgment was superior to EWS in predicting ICU admission rather than death is consistent with this interpretation (Tables S4 and S5).

A second explanation involves differences in the types of information integrated by each approach. Triage systems require recognition of clinical syndromes that are not solely expressed through vital sign derangement. Some conditions may initially present with near‐normal vital parameters yet still warrant high‐dependency or intensive care. Clinical judgment also incorporates contextual information, including caregivers' perceptions of baseline status, collateral histories from pre‐hospital personnel, and implicit assessments of dynamic trajectories, none of which are captured by single‐time‐point vital signs. Supporting this view, recent findings suggest that, while artificial intelligence tools can match physician judgment, the combination of both approaches may provide superior performance [38]. This consideration also highlights the gap between prediction and decision, with triage evaluation being an example of the latter. In this sense, clinical judgment at triage can arguably be viewed more as a decision tool, rather than a mere prediction tool. Triage decisions are aimed at identifying patients who are most likely to benefit from escalation of care, rather than patients with higher unmodifiable risk. Consequently, triage decisions take into account the actionability of a prediction, in addition to its accuracy. This interpretation is supported by the relative advantage achieved, in our data, by clinical judgment compared to EWS in predicting ICU admission, rather than death (Tables S4 and S5). Thus, our data could support the idea that clinical judgment might predict acuity rather than severity, that is, the need for time‐critical intervention rather than the overall risk of adverse outcome. The smaller differences observed between clinical judgment and EWS in predicting the composite outcome of ICU and death might underestimate the ability of the former to identify patients where the risk of death is modifiable by escalation of care. This would align with the idea that using a tool devised to optimize accuracy on prognostic outcomes (like death) may lead to selecting a larger proportion of patients unlikely to benefit from an intervention [39, 40].

Indeed, a recent study found that the NEWS2 showed unsatisfactory performance in identifying patients needing time‐critical intervention in the ED setting, with half of them not reaching the standard NEWS2 threshold [41]. Interestingly, many of the false negatives presented with conditions which have no immediate consequence on vital signs and whose identification requires a broader appreciation of the patient's clinical picture.

A third consideration is that widespread implementation of structured decision frameworks may influence unstructured judgment. Healthcare professionals routinely exposed to decision rules may incorporate their cues into clinical judgment, consciously or unconsciously. Comparisons between decision rules and unstructured judgment may therefore be inherently biased because the latter may already embed structured rule‐derived information. Clinical judgment and structured rules can thus be seen as co‐evolving systems. Judgment may highlight clinically relevant cues later formalized in rules, while rules may emphasize predictors that clinicians may previously have underestimated.

Finally, drawing on concepts from decision science, triage may align more closely with a “kind learning environment” rather than a “wicked” one [42, 43]. In kind learning environments, stable conditions, repeated exposure to comparable scenarios, and rapid and accurate feedback enable healthcare professionals to refine their unstructured judgment over time. Under these circumstances, practitioners may learn to detect consistent relationships between cues and outcomes even when these are not explicitly formalized in algorithmic models.

## 5 Limitations

The study has several limitations. It was conducted at a single urban academic center, which may limit its generalizability. The retrospective design introduces potential inaccuracies in data capture and the possibility of unmeasured confounding. Clinical judgment was approximated using the triage category, which represents the endpoint of a complex and dynamic process. Because triage systems require vital sign measurement, clinical judgment and EWS share overlapping physiological inputs, which may allow for some degree of information leakage. Nevertheless, this design reflects real‐world operations and ensures that the comparison mirrors routine clinical practice. We also lacked complete data on overall prognosis and advanced directives, both of which may influence triage decisions and downstream outcomes. We did not exclude repeated encounters by the same patient over time, which may introduce within‐patient correlation between observations. However, given the large sample size and the high volume of ED visits, this dependency is unlikely to meaningfully affect the overall results. Moreover, we chose a 24 h follow‐up time. While consistent with the existing literature on the use of EWS in the ED setting, this choice may have overlooked potential events occurring beyond this time frame. A large proportion of patients (30%) was excluded from the final analysis because they were discharged or transferred to lower‐intensity care before the prespecified 24 h time frame. While no data were available on the outcome of these patients, they are likely to represent a subgroup with reduced risk of adverse outcome. Consequently, their exclusion may have enriched the analytic cohort for higher‐risk patients and prevented the capture of early adverse events occurring after discharge, including readmission or presentation to other hospitals, potentially affecting performance estimates. Finally, our study assessed predictive accuracy only for early adverse events, leaving the question of actual patient‐centered benefit largely untouched. Importantly, improved predictive accuracy does not automatically translate into improved patient outcomes. A recent evaluation of an automated ED prediction tool, for example, showed superior predictive accuracy compared with clinicians, yet failed to demonstrate improvements in patient‐relevant endpoints [44].

## 6 Conclusions

In conclusion, our findings indicate that existing EWS did not clearly outperform the nuanced, partially structured, or unstructured expert judgment applied during ED triage. Notably, clinical judgment might have outperformed EWS in identifying patients with a modifiable clinical trajectory. Future investigations should evaluate this latter possibility, explore the degree of overlap between EWS and clinical judgment, and identify clinical contexts in which their predictions diverge. A better understanding of when each approach provides greatest clinical value may support more effective integration of structured scores with clinical judgment in routine acute care.

## Author Contributions

M.C. and D.A.D.P. conceived the study.M.C., D.A.D.P., and P.M.C.F. collected and analyzed the data. P.M.C.F. provided statistical expertise. M.C., D.A.D.P., P.M.C.F., and N.B. interpreted the results. D.A.D.P., N.B., and V.M. drafted the manuscript. M.C., C.S., A.G., F.F., A.P., and G.D.M. critically revised the manuscript for important intellectual content.

## Funding

The authors have nothing to report.

## Conflicts of Interest

The authors declare no conflicts of interest.

## Supporting information


**Figure S1:** Calibration plot of EWS models and clinical judgment.
This plot compares the predicted probabilities and observed outcome frequencies for five early warning score (EWS) and clinical Judgment logistic regression models. The dashed diagonal line represents perfect calibration (slope = 1), where predicted risk matches observed risk. A well‐calibrated model produces points that lie close to the diagonal line in the calibration plot and, consistently, a lower Brier score. Conversely, systematic deviations from the diagonal, particularly in more frequent risk regions.
**Table S1:** Calibration in the large. CITL (Calibration‐in‐the‐Large) is a calibration metric that assesses whether a predictive model systematically overestimates or underestimates outcomes. It represents the difference between the average predicted risk and the average observed outcome. A CITL of zero indicates perfect alignment; a positive value means overprediction, and a negative value means underprediction. The table shows that all the models, except NEWS and NEWS2, had a negative CITL value (underprediction). However, no significant difference in calibration emerged among the evaluated EWSs and the Clinical Judgment
**Table S2:** Brier score. The table reports the Brier score (with 95% CI) for each model, along with its variation compared with the null model (based on prevalence) and with clinical assessment, including the corresponding *p*‐values. The Brier score measures the overall accuracy of predicted probabilities by combining discrimination and calibration; lower values indicate better performance. All models (Clinical Assessment and EWS) show a significant improvement over the null model (negative ΔBrier, *p* < 0.001), indicating that they provide real predictive information beyond prevalence alone. Clinical assessment has the lowest Brier score, representing the best reference in terms of global probabilistic accuracy. All EWS have a slightly worse Brier score compared with clinical assessment (positive ΔBrier vs. Clinical Assessment, *p* < 0.001), suggesting that although they perform significantly better than the null model, they do not surpass the overall probabilistic performance of clinical assessment.
**Table S3:** Net reclassification improvement (NRI). The Net Reclassification Index (NRI) measures whether a new prediction model improves the correct classification of individuals, compared with an existing model, by reassigning them to more appropriate risk categories. The table shows the NRI values vs. Clinical Judgment. All the EWS had a negative NRI, indicating a worse trade‐off between risk assigned and events. However, the differences did not reach statistical significance.
**Figure S2:** Radar plots comparing clinical assessment and early warning scores at matched specificity thresholds. Specificity was fixed at the level of Clinical Judgment “urgency” (Probability threshold used 0.00151), and corresponding thresholds for each early warning score were identified. Sensitivity, PPV, NPV, and likelihood ratios were then calculated at these thresholds, with LR+ and LR− normalized to a 0–1 range for comparability across metrics. In clinical risk prediction with a low event rate, overtriage (too many false alarms) is a major concern. Models may detect most true cases (high sensitivity) but mistakenly flag large numbers of low‐risk patients as “high risk” (low specificity), overwhelming resources. Therefore, it is often clinically useful to fix specificity at a realistic, acceptable level, and then compare models based on how much sensitivity they achieve at that specificity. In our analysis, we identified the specificity for each class of Clinical Judgment; then, the same specificity was identified for the EWS scores, and the remaining performance parameters were compared in the figure.
**Figure S3:** Radar plots comparing clinical assessment and early warning scores at matched specificity thresholds. Specificity was fixed at the level of Clinical Judgment “Emergency” (Probability threshold used 0.160), and corresponding thresholds for each early warning score were identified. Sensitivity, PPV, NPV, and likelihood ratios were then calculated at these thresholds, with LR+ and LR− normalized to a 0–1 range for comparability across metrics. In clinical risk prediction with a low event rate, overtriage (too many false alarms) is a major concern. Models may detect most true cases (high sensitivity) but mistakenly flag large numbers of low‐risk patients as “high risk” (low specificity), overwhelming resources. Therefore, it is often clinically useful to fix specificity at a realistic, acceptable level, and then compare models based on how much sensitivity they achieve at that specificity. In our analysis, we identified the specificity for each class of Clinical Judgment; then, the same specificity was identified for the EWS scores, and the remaining performance parameters were compared in the figure.
**Table S4:** ROC curve analysis comparison of Clinical Judgment vs. the Early warning scores, for the prediction of death within 24 h. The best performer was VIEWS, which had similar discrimination compared to REMS. Clinical Judgment and NEWS/NEWS2 had significantly lower prediction results.
**Table S5:** ROC curve analysis comparison of Clinical Judgment vs. the Early warning scores, for the prediction of admission to ICU within 24 h. The best performer was the Clinical Judgment, which outperformed all the EWS.

## Data Availability

The data that support the findings of this study are available from the corresponding author upon reasonable request and after approval by the local Institutional Review Board.
