# Supplementary material for: Assessing Risk of Early ICU Admission or Death at Emergency Department Triage: Clinical Judgment Versus Early Warning Scores
Source: Acad Emerg Med. 2026 Jul 16;33(7):e70377. doi: 10.1111/acem.70377 (PMC13374591; doi:10.1111/acem.70377)
Supplement: Supplementary file 1 — Figure S1: Calibration plot of EWS models and clinical judgment. This plot compares the predicted probabilities and observed outcome frequencies for five early warning score (EWS) and clinical Judgment logistic regression models. The dashed diagonal line represents perfect calibration (slope = 1), where predicted risk matches observed risk. A well‐calibrated model produces points that lie close to the diagonal line in the calibration plot and, consistently, a lower Brier score. Conversely, systematic deviations from the diagonal, particularly in more frequent risk regions. Table S1: Calibration in the large. CITL (Calibration‐in‐the‐Large) is a calibration metric that assesses whether a predictive model systematically overestimates or underestimates outcomes. It represents the difference between the average predicted risk and the average observed outcome. A CITL of zero indicates perfect alignment; a positive value means overprediction, and a negative value means underprediction. The table shows that all the models, except NEWS and NEWS2, had a negative CITL value (underprediction). However, no significant difference in calibration emerged among the evaluated EWSs and the Clinical Judgment Table S2: Brier score. The table reports the Brier score (with 95% CI) for each model, along with its variation compared with the null model (based on prevalence) and with clinical assessment, including the corresponding p‐values. The Brier score measures the overall accuracy of predicted probabilities by combining discrimination and calibration; lower values indicate better performance. All models (Clinical Assessment and EWS) show a significant improvement over the null model (negative ΔBrier, p < 0.001), indicating that they provide real predictive information beyond prevalence alone. Clinical assessment has the lowest Brier score, representing the best reference in terms of global probabilistic accuracy. All EWS have a slightly worse Brier score compared with clinical assessme [file ACEM-33-0-s001.docx]

**Supplementary Figure 1** - Calibration Plot of EWS Models and Clinical Judgment.
This plot compares the predicted probabilities and observed outcome frequencies for five Early Warning Score (EWS) and clinical Judgment logistic regression models. The dashed diagonal line represents perfect calibration (slope = 1), where predicted risk matches observed risk.

A well-calibrated model produces points that lie close to the diagonal line in the calibration plot and, consistently, a lower Brier score. Conversely, systematic deviations from the diagonal, particularly in more frequent risk regions


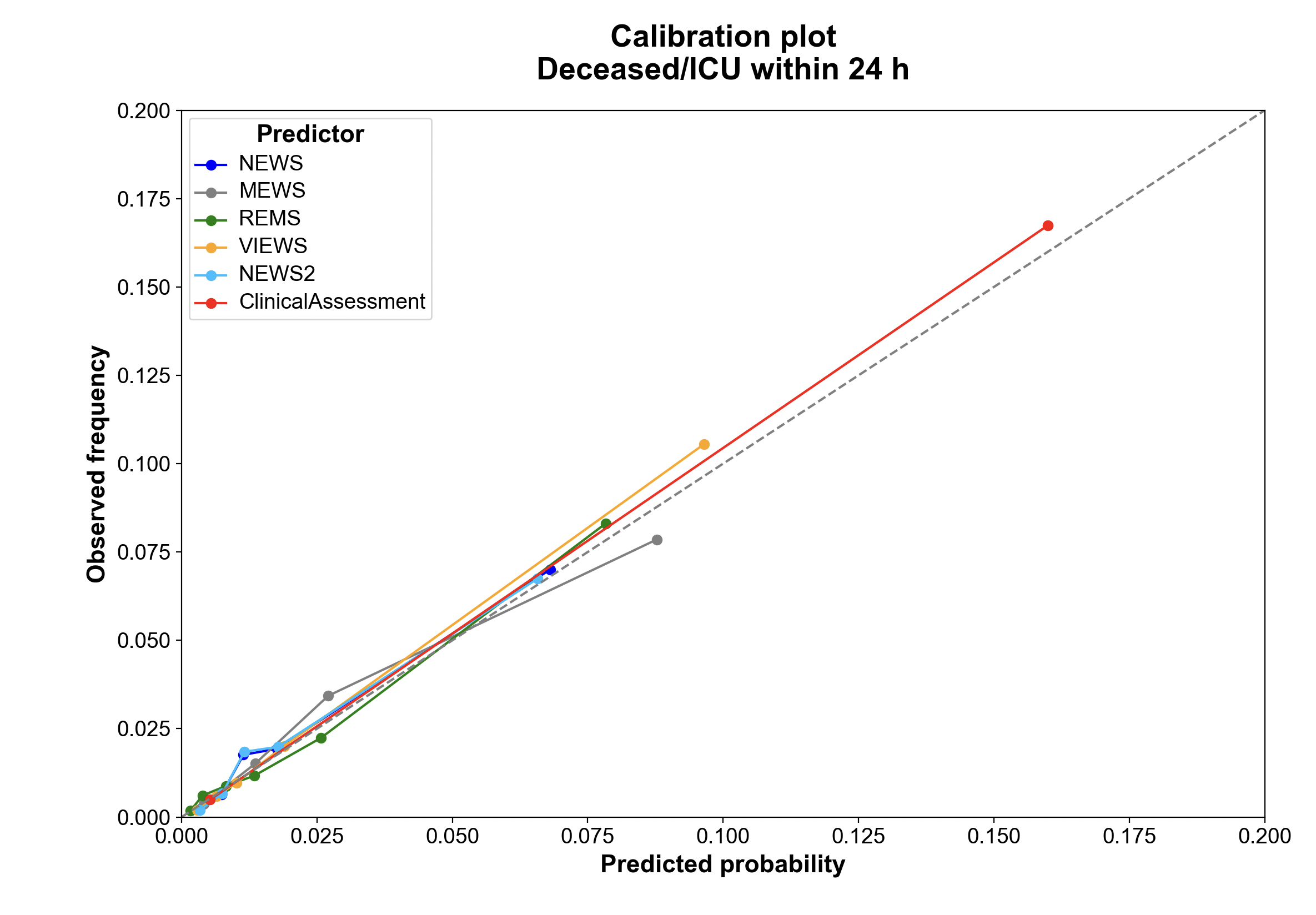


**Supplementary Table 1 – Calibration in the Large**

CITL (Calibration-in-the-Large) is a calibration metric that assesses whether a predictive model systematically overestimates or underestimates outcomes. It represents the difference between the average predicted risk and the average observed outcome. A CITL of zero indicates perfect alignment; a positive value means overprediction, and a negative value means underprediction.

The table shows that all the models, except NEWS and NEWS2, had a negative CITL value (underprediction). However, no significant difference in calibration emerged among the evaluated EWSs and the Clinical Judgment

| **Predictor** | **CITL (95% CI)** | **Slope (95% CI)** | **p-value CITL vs Clinical Judgment** | **p-value Slope vs Clinical Judgment** |
| --- | --- | --- | --- | --- |
| **NEWS** | 0.001 [-0.053-0.065] | 1.001 [0.984-1.020] | 0.907 | 0.858 |
| **MEWS** | -0.003 [-0.085-0.074] | 1.000 [0.978-1.018] | 0.963 | 0.904 |
| **REMS** | -0.001 [-0.096-0.102] | 1.000 [0.976-1.024] | 0.937 | 0.902 |
| **VIEWS** | -0.007 [-0.078-0.060] | 0.998 [0.982-1.014] | 0.989 | 0.989 |
| **NEWS2** | 0.002 [-0.096-0.094] | 1.000 [0.979-1.019] | 0.904 | 0.877 |
| **Clinical Judgment** | -0.006 [-0.071-0.052] | 0.998 [0.981-1.018] | — | — |

**Supplementary Table 2 – Brier Score**

The table reports the Brier score (with 95% CI) for each model, along with its variation compared with the null model (based on prevalence) and with clinical assessment, including the corresponding p-values. The Brier score measures the overall accuracy of predicted probabilities by combining discrimination and calibration; lower values indicate better performance.

All models (Clinical Assessment and EWS) show a significant improvement over the null model (negative ΔBrier, p < 0.001), indicating that they provide real predictive information beyond prevalence alone. Clinical assessment has the lowest Brier score, representing the best reference in terms of global probabilistic accuracy. All EWS have a slightly worse Brier score compared with clinical assessment (positive ΔBrier vs Clinical Assessment, p < 0.001), suggesting that although they perform significantly better than the null model, they do not surpass the overall probabilistic performance of clinical assessment.

| **Predictor** | **Intercept (CI)** | **Regression Coeff. (CI)** | **OR (CI)** | **ROC-AUC (CI)** |
| --- | --- | --- | --- | --- |
| **NEWS** | -5.782 [-5.837--5.728] | 0.441 [0.432-0.450] | 1.554 [1.540-1.568] | 0.857 [0.851-0.862] |
| **MEWS** | -5.686 [-5.739--5.632] | 0.702 [0.686-0.717] | 2.017 [1.986-2.049] | 0.818 [0.812-0.824] |
| **REMS** | -6.791 [-6.873--6.709] | 0.416 [0.406-0.426] | 1.515 [1.500-1.531] | 0.813 [0.807-0.818] |
| **VIEWS** | -5.919 [-5.975--5.862] | 0.445 [0.437-0.454] | 1.561 [1.548-1.574] | 0.875 [0.870-0.880] |
| **NEWS2** | -5.753 [-5.807--5.699] | 0.436 [0.427-0.445] | 1.547 [1.533-1.560] | 0.853 [0.847-0.859] |
| **Clinical Judgment** | -6.693 [-6.772--6.614] | 2.517 [2.468-2.567] | 12.394 [11.794-13.026] | 0.872 [0.866-0.877] |

**Supplementary Table 3 – Net Reclassification Improvement (NRI)**

The Net Reclassification Index (NRI) measures whether a new prediction model improves the correct classification of individuals, compared with an existing model, by reassigning them to more appropriate risk categories. The table shows the NRI values vs Clinical Judgment. All the EWS had a negative NRI, indicating a worse trade-off between risk assigned and events. However, the differences did not reach statistical significance.

| **Model** | **NRI Events (95% CI)** | **NRI Non-Events (95% CI)** | **NRI Total (95% CI)** |
| --- | --- | --- | --- |
| **NEWS** | -0.22 (-0.25--0.19) | -0.50 (-0.50--0.49) | -0.72 (-0.74--0.69) |
| **MEWS** | -0.38 (-0.41--0.35) | -0.46 (-0.47--0.46) | -0.84 (-0.87--0.82) |
| **REMS** | -0.32 (-0.35--0.29) | -0.07 (-0.07--0.06) | -0.39 (-0.42--0.36) |
| **VIEWS** | -0.16 (-0.19--0.13) | -0.50 (-0.50--0.50) | -0.66 (-0.69--0.63) |
| **NEWS2** | -0.24 (-0.27--0.21) | -0.50 (-0.50--0.50) | -0.74 (-0.77--0.71) |

**Supplementary Figure 2**

Radar plots comparing clinical assessment and early warning scores at matched specificity thresholds. Specificity was fixed at the level of Clinical Judgment “urgency” (Probability threshold used 0.00151), and corresponding thresholds for each early warning score were identified. Sensitivity, PPV, NPV, and likelihood ratios were then calculated at these thresholds, with LR+ and LR− normalized to a 0–1 range for comparability across metrics.

In clinical risk prediction with a low event rate, overtriage (too many false alarms) is a major concern. Models may detect most true cases (high sensitivity) but mistakenly flag large numbers of low-risk patients as “high risk” (low specificity), overwhelming resources. Therefore, it is often clinically useful to fix specificity at a realistic, acceptable level, and then compare models based on how much sensitivity they achieve at that specificity. In our analysis, we identified the specificity for each class of Clinical Judgment; then, the same specificity was identified for the EWS scores, and the remaining performance parameters were compared in the figure.


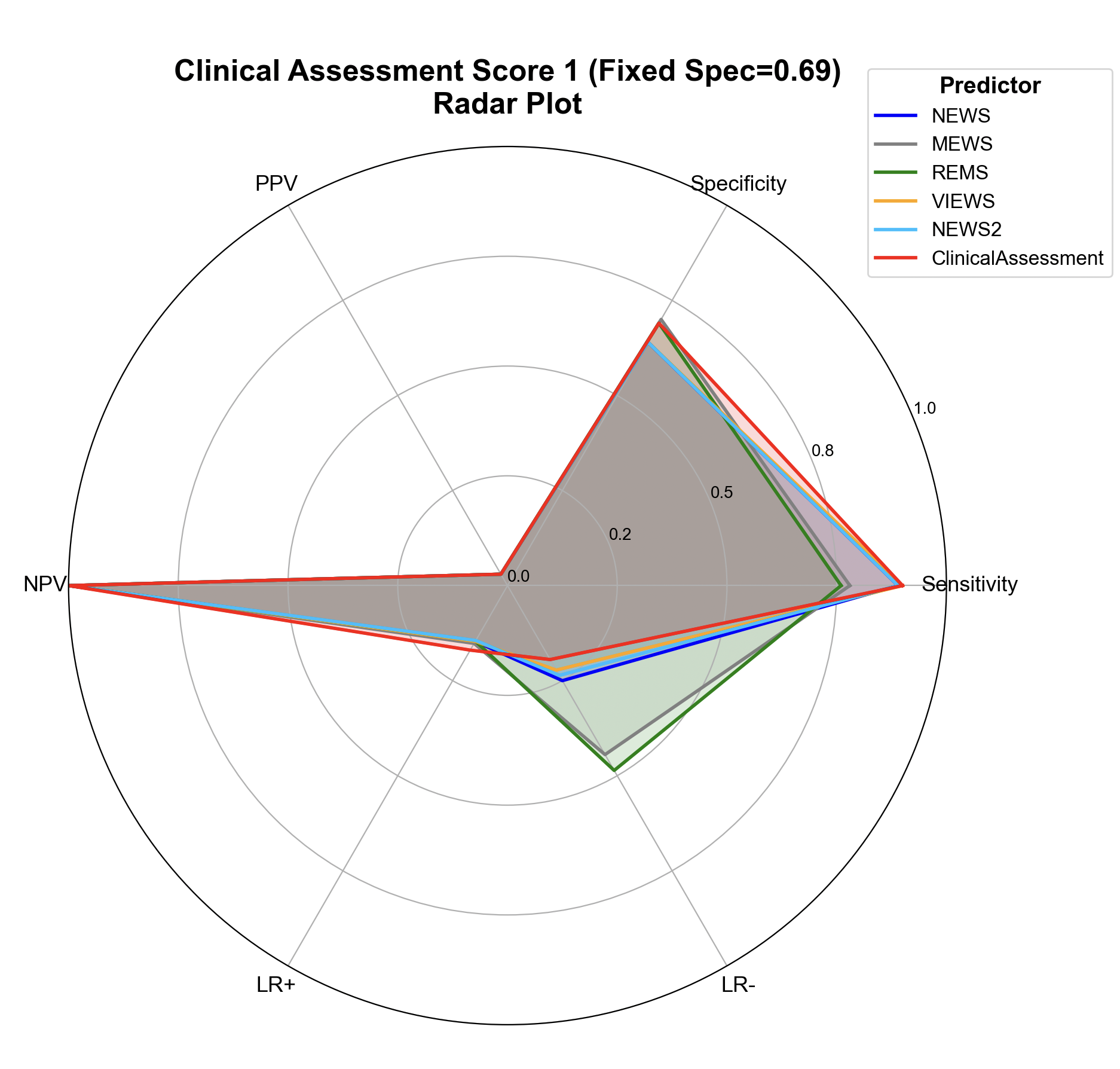


**Supplementary Figure 3**

Radar plots comparing clinical assessment and early warning scores at matched specificity thresholds. Specificity was fixed at the level of Clinical Judgment “Emergency” (Probability threshold used 0.160), and corresponding thresholds for each early warning score were identified. Sensitivity, PPV, NPV, and likelihood ratios were then calculated at these thresholds, with LR+ and LR− normalized to a 0–1 range for comparability across metrics.

In clinical risk prediction with a low event rate, overtriage (too many false alarms) is a major concern. Models may detect most true cases (high sensitivity) but mistakenly flag large numbers of low-risk patients as “high risk” (low specificity), overwhelming resources. Therefore, it is often clinically useful to fix specificity at a realistic, acceptable level, and then compare models based on how much sensitivity they achieve at that specificity. In our analysis, we identified the specificity for each class of Clinical Judgment; then, the same specificity was identified for the EWS scores, and the remaining performance parameters were compared in the figure.


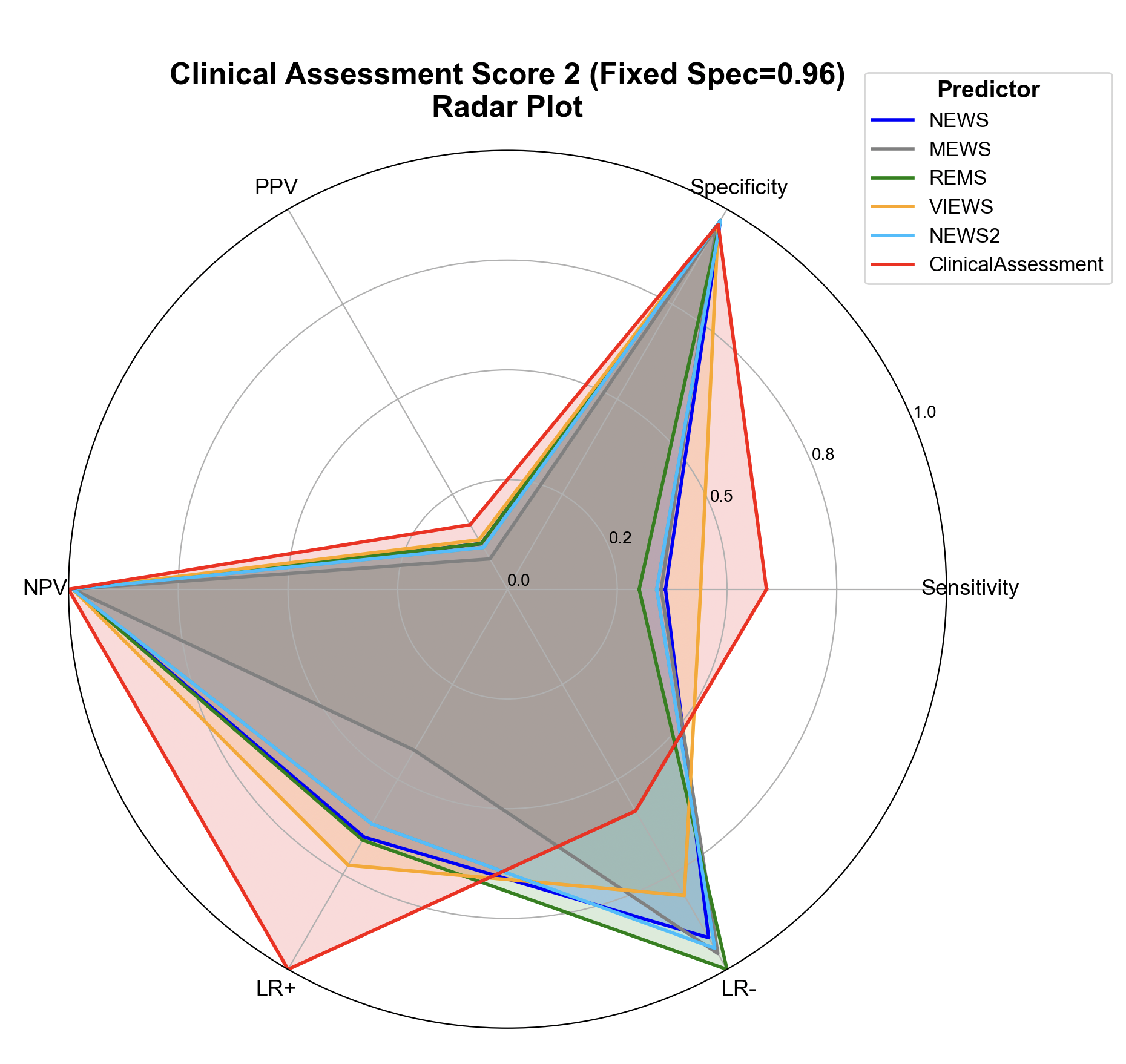


**Supplementary Table 4**

ROC curve analysis comparison of Clinical Judgment vs. the Early warning scores, for the prediction of death within 24 hours. The best performer was VIEWS, which had similar discrimination compared to REMS. Clinical Judgment and NEWS/NEWS2 had significantly lower prediction results.

| **Score** | **ROC AUC [95% CI]** | **DeLong  p-value** |
| --- | --- | --- |
| **Clinical Judgment** | 0.851 [0.840-0.862] | <0.001 |
| **NEWS** | 0.882 [0.873-0.891] | 0.0053 |
| **NEWS2** | 0.880 [0.871-0.889] | 0.0074 |
| **MEWS** | 0.844 [0.833-0.854] | <0.001 |
| **REMS** | 0.883 [0.876-0.891] | 0.4782 |
| **VIEWS** | 0.887 [0.877-0.896] | 1 |

**Supplementary Table 5**

ROC curve analysis comparison of Clinical Judgment vs. the Early warning scores, for the prediction of admission to ICU within 24 hours. The best performer was the Clinical Judgment, which outperformed all the EWS.

| **Score** | **ROC AUC [95% CI]** | **DeLong  p-value** |
| --- | --- | --- |
| **Clinical Judgment** | 0.879 [0.871-0.886] | 1 |
| **NEWS** | 0.844 [0.837-0.851] | <0.001 |
| **NEWS2** | 0.839 [0.832-0.846] | <0.001 |
| **MEWS** | 0.806 [0.768-0.785] | <0.001 |
| **REMS** | 0.776 [0.768-0.785] | <0.001 |
| **VIEWS** | 0.868 [0.861-0.875] | 0.0077 |
